# Milk Secreted by Tumors in the Axillœ

**Published:** 1860-10

**Authors:** Juriah Harriss

**Affiliations:** Professor of Physiology in the Savannah Medical College.


### ARTICLE XIX.

*Milk Secreted by Tumors in the Axillæ.* By Juriah Harriss, M. D., Professor of Physiology in the Savannah Medical College.

This case, so far as my reading extends, presents unusual and interesting features, and is in some respects unique. I will first present the case, and follow its report by remarks and explanations of the anomalous features, as seem to me most rational and physiological.

The patient came under the observation of my friend, Dr. J. Clay Habersham, of this city. During the spring, Dr. H. handed me a four ounce vial containing a rich yellowish white fluid so much resembling human milk that I at once supposed it must be such, though I had not learned from whence it came. The Dr. requested me to make a microscopical examination, but time did not then permit, and I handed it to my colleague, Prof. Pratt, for analysis. I, however, subsequently made an examination, and found abundant milk and a few colostrum corpuscles.

The following is a history of the case:

A negro woman, Amy, aged about 38 years, well formed, stout and healthy, and of healthy family—mother of eight children, five of whom are now living. She has been perfectly healthy both as a girl and a married woman. When she gave birth to the third child, she experienced great pain in and about the right shoulder and numbness of hand. This pain was supposed by the attending physician to be of rheumatic character. She was accordingly treated for this affection, blistered, &c.

Soon after commencing to nurse the third child, she noticed small tumors, about the size of pigeons' eggs, in each axilla. She does not recollect to what size they grew, during that period of lactation, nor is she sure that there was any increase in volume after lactation had commenced. She is positive, however, that the tumors did not diminish after she weaned the third child, and the milk ceased to flow from the mammæ. During the pregnancy with fourth child, the tumors were not further developed, but after the birth of the fourth child, and at period of commencing lactation, there was an increased development of the tumors, attended with the same sensation of distension and hardness that she experienced in the mammæ. She thinks, but is not certain, that it was after the birth of the fifth child that she drew the attention of Dr. Habersham, (father of Dr. J. Clay Habersham,) to these "swellings." He deemed it necessary to tap both of these tumors, and as was recently obtained; about one pint from the right did so, drawing off about the same quantity of similar fluid tumor, and much less from the left.

The tumors did not again enlarge, until the birth of the sixth child. Again she experienced the same sensations in the tumors as in the mammæ, just previous to the secretion of the milk, when they became larger; and when milk appeared in the mammary glands, the tumors were soft but more distended than after birth of last child—they were not tapped during this lactation. They remained the same size during this period, and did not subside after the child was weaned. They enlarged at each period of commencing lactation, or were at least more distended and inconvenient. This spring, and during the nursing of the eighth child, Dr. J. C. Habersham's attention was drawn to the tumors. He found the right tumor the larger and more fluctuating one. He at once tapped it and gave me the fluid to examine. He drew about a pint, all that the sac contained. About a week after, I rode with him to the rice plantation, a few miles from town, in order to examine the tumors. I found the tumor in right axilla, the one emptied by trochar, very flaccid loose tegumentary structure, having grown of elliptical form, with no contents. The surfaces flattened were those pressed on by the arm and the chest. At the base of the tumor and in the axilla, could be felt a lobulated and hardened structure. Indeed the tumor resembled in appearance and to the touch, a flaccid mamma which had ceased to secrete milk, with the exception that there was not even a rudimentary nipple existing.

The tumor in the left axilla was smaller and could not contain as much fluid as that of the right. It was, however, about the size of a large orange, no appearance of a nipple; fluctuation very apparent by percussion. I punctured it with a trochar, and drew off about an ounce of the milky fluid, but this did not empty the tumor entirely, as upon the outer side could still be felt fluctuation. The side tapped became like the tumor of the right side, flaccid, but the other portion contained evidently a fluid. This would prove that the tumor of the left was multilocular, while that of the right was unilocular. But one of the sacs of the left was emptied. Dr. H. informs me that both tumors are still in the same condition, though three months have passed, and the mother nursing the child. There has been no accumulation of fluid since.

The child was fourteen months of age at the time the tumors were last punctured. The woman states that between the periods of tapping, from fourth to eighth child the tumors were less distended after than during lactation, but never became flaccid, as when tapped.

The point of greatest interest in this case, is how did the milky fluid, possessing the physical and chemical characteristics of milk, find its way into the sacs of the axillæ.

I have diligently searched numerous works and find no reference to such a state of things, and no parallel case recorded. I have looked through works, not a few, upon physiology, and pathological anatomy, but have seen no reference to a similar anomalous arrangement. Cruveilhier, a large and beautifully illustrated work, contains nothing connected with this subject.

But three hypotheses suggest themselves to my mind in explanation of this anomaly. 1st. That the milk secreted by the mammary glands has been absorbed and conveyed to the axillæ by blood vessels, or absorbents proper, or a communication of lacteal ducts extending from mammary to axillary glands. 2d. That the fluid was secreted by the lymphatic glands of this locality. 3d. That there was in each axilla a morphological *nisus*, developing a mammary glandular tissue, which secreted the milky fluid.

The first supposition that the milk has been absorbed and conveyed from mammæ to axillæ, is utterly untenable, inasmuch as it is physically impossible for milk corpuscles, and more especially for colostrum corpuscles to pass through the pores of blood vessels, or lymphatics, and it has never been proven that the latter vessels have open mouths, as was formerly contended that the lacteals had. There is here then a denial of such a possibility from the physical structure of the absorbent tissue.

The second supposition is equally untenable, that the lymphatic glands of the axillæ took on the office of secreting milk. This view is in contradiction to the physiological laws now accepted, as applying to secretions. The secreting property of each gland or membrane, exists in the epithelial cells lining the ducts of glands or the surface of membranes, which possess the vital property of selecting elements from the blood, which form the fluid peculiar to each gland or secreting membrane. I know of no author who denies the property of such cells, as a universal law, save Dr. Draper. He, strange to say, admits the law as gonerally applicable to secreting tissues, but makes the liver an exception to the law. That is, that all other secreting tissues have cells peculiar to themselves, possessing the property of separating certain elements, which when combined form certain secretions belonging to certain secreting tissues. I will not, as it is too great a digression, discuss this chemico-physiological view of the subject. Suffice it to say, that Dr. Draper has given no satisfactory explanation of this exceptional violation of the general, and as I believe, universal law of the organism. That some secreting tissues do partially take on, under certain pathological conditions, the office of other tissues, is unquestioned and no longer denied by authorities. But there is no clear proof that there is an *entire* substitution of the office of one gland for the other. The nearest approach to a substitutive action of one gland for another is one (the chemical analysis of the fluid secreted by the kidneys,) may be found in the first volume of the *Sav. Journal of Medicine*, accompanied by editorial remarks. In that case the fluid was drawn from the bladder with the catheter, by the attending physician, so that no deception could have been practiced. In the editorial remarks in connection with that case, I repudiate the idea that one gland can secrete a fluid in its physiological or pathological state, with normal elements and normal proportions of the component parts which form the secretion of another glandular structure. I there made use of the following language: "That one secreting gland can take on the normal function of another in *every respect*, we are loth to admit, believing as we do that each gland has secreting cells peculiar to itself, possessing distinct and peculiar properties." "But it is well proven that some glands do take on a substitutive action for another to a considerable extent."

The third is the explanation I believe most plausible, viz. that in this instance there existed in the axillæ, a morphological *nisus*, as there always is at the situation of the mammary glands. That the morphological change, or the development of mammary secreting tissue was simultaneous in mammæ proper and in the axillæ—the latter to a much less extent. In this event, the tumors of axillæ are but *supplementary mammary glands*, somewhat rudimentary in form. The history of the case, the gradual and stated periods of enlargement, and the sensations accompanying the enlargement, being not only identical in character, but existing simultaneously with those in mammæ when milk is first secreted, are proofs, aside from the peculiar form of sacs and lobulated masses at the base of the tumors, that there was a development of mammary glandular structure. The axillary tumors were, so far as appearances and palpation could serve as guides, entirely separate and distinct—there seemed to be no connection whatever. But why should the tumors not have been developed and milk secreted during the first and second lactation, as the subsequent history of the case proves did occur, from the third to the eighth lactation inclusive? The amount and imperfect development of the mammary tissue in axillæ was doubtless small in amount, and rudimentary in character, thus requiring several impregnations and lactations to stimulate its growth and secreting powers. It should be remembered that the tumors, about the size of pigeons eggs, were first noticed during the third pregnancy, and were largely developed at the third lactation, accompanied by a feeling of fullness and pain, identical in character to that felt in the mammæ at the commencement of the third and succeeding lactations. No growth nor peculiar sensations, were felt save at the time of enlargement and distention of mammæ at commencement of lactation.

My colleague, Prof. Arnold, states that he witnessed in a servant girl, now living, three mammary glands. In this case there seemed to be a division of one of the glands, which formed the third, each of the three have nipples.

---

Dr. Juriah Harriss—

*Dear Sir:*—In accordance with your request, I have examined the contents of the tumor lanced by Dr. J. C. Habersham on the 16th inst., and herewith send a minute description of the same.

The fluid when first examined, had an acid reaction and seemed to be in a semi-coagulated condition, a slight creamy layer floated on the yellowish milky fluid below. I could detect *no sedimentarg deposit.*

Under the microscope, I at once recognized the *fat globules of milk,* and scattered through the field of view, appeared the very characteristic *colostrum corpuscles,* conglomerates of fine fatty molecules aggregated together by means of a hyaline substance scarcely perceptible. The appearance of the field was remarkably similar to the figure given by Funke in his Atlas.* The fatty globules

* Atlas of Physiological Chemistry, by Dr. Otto Funke, Cavendish Society Publications, London.

ran together action of acetic acid, dissolving the albuminous envelop.

Although specially looked for, *pus corpuscles could not be found.*

By chemical examination, *the entire absence of pus* was confirmed, and the presence of all the constituents of colostrum and milk ascertained. On account of the semi-coagulated condition of the fluid, caused by its acidity, I cannot say positively whether the nitrogenous matter present was albumen or casein, though from the deportment of the fluid during evaporation, I suspect the presence of both.

I append, for convenience of reference, a comparative table of the composition in 1000 parts of 1st, Colostrum. 2d, Average of normal milk. 3d, Contents of axillary tumor.

| | Colostrum. | | Milk. | | Tumor. | |
|---|---|---|---|---|---|---|
| Water, . . . . . . | | 828.00 | | 891.00 | | 878.80 |
| Albuminous matter, . . | 40.00 | (Casein) | 33.70 | | 35.02 | |
| Fatty matter, (butter,) . . | 50.00 | | 37.10 | | 39.08 | |
| Sugar of milk, . . . . | 70.00 | | 38.50 | | 43.50 | |
| Salts, . . . . . . | 3.10 | 172.00 | 1.89 | 109.00 | 3.60 | 121.20 |

The specific gravity of the milk was 1025.

It will be observed that in composition the tumor stands intermediate between colostrum and normal milk, with the single exception of the quantity of salt.

Very respectfully, yours,

N. A. PRATT,

[*Sav. Jour. of Med.*

